# Criminal Careers in the Making? Characteristics and Trajectories of Juveniles Charged With a Sexual Offense

**DOI:** 10.1177/10790632251393988

**Published:** 2025-11-05

**Authors:** Christine Friestad, Torbjørn Skardhamar

**Affiliations:** 1 Department of Mental Health and Addiction, Centre of Forensic Education and Research, 155272 Oslo University Hospital, Oslo, Norway; 2 6268 University College of Norwegian Correctional Service, Lillestrøm, Norway; 3 Department of Sociology and Human Geography, 6305 University of Oslo, Norway

**Keywords:** adolescent sexual abusers, criminal recidivism, juvenile sex offender recidivism, sexual recidivism

## Abstract

The present study investigates the onset, persistence and diversity in sexual offending by exploring Norwegian registry data on the social and criminal trajectories of juveniles charged with a sexual crime before the age of criminal responsibility, compared to juveniles charged with other types of offenses. The sample consisted of all persons aged 15 during the period 1997–2005 (*N* = 388,814). Of those, 19,559 juveniles (5%) were charged with a crime, mostly (83%) of a non-violent nature. For the remaining 16.9% (*n* = 3,309), the charge involved violence, either non-sexual (*n* = 2,991, 15.3% of those charged) or sexual (*n* = 318, 1.6% of those charged). Proportional hazard models were used to investigate background characteristics and risk of recidivism. Early onset of offending was related to a more disadvantaged background. Juveniles charged with violent and sexual offenses were generally younger and had more charges against them than juveniles charged with any other crimes. Early sexual crime carried higher hazard rate (HR) of future sexual crime (HR = 3.6) than early violent (HR = 1.9) and other crimes (HR = 1.4). The risk of later violent crime was similar among those with early onset violent (HR = 2.8) and sexual (HR = 2.5) crime, and higher than for early onset general crime (HR = 1.7). Since violent crime has a higher base rate than sexual crime, violence is more dominant in the criminal careers of people with early onset sexual offending. However, the majority of adult sexual criminal charges are raised against persons without a juvenile criminal record.

## Introduction

Despite decades of research, fundamental empirical questions remain related to whether and how sexual offending at a young age is indicative of a life-course pattern of criminal involvement (Lussier et al., 2024). The question of (dis-)continuity in offending from childhood/youth to adulthood lies at the heart of a criminal career approach, which is still relatively new to the sexual offending field (see Blokland & Lussier, 2015; Cale & Lussier, 2014; Lussier & Cale, 2013). Thus, it remains an open question whether perpetrators of sexual offending are similar to perpetrators of other crimes, and whether sexual offending unfolds over time in a fashion similar to non-sexual offending (Lussier et al., 2021).

### Sexual Offending as Part of a Criminal Career

As noted by Lussier and coworkers (2021) criminal career research focuses on a person’s *behavior*, that is, the sexual offending itself. Clinical research on sexual offending has mostly focused on individual (cognitive, emotional, relational) characteristics related to what is assumed to be a stable and fixed propensity to commit sexual crimes. Approaches that focus on deconstructing the behavior in question (sexual offending) into its constituent parts (Lussier & Cale, 2013) represent an important addition to the knowledge base. Criminal career research focuses on the longitudinal sequencing of offending patterns (cf. Blumstein & Cohen, 1987), and has provided a set of parameters (onset, frequency, continuity, diversity, desistance, etc.) to break down the development of offending over time into parts that can be subject to empirical investigation (see Piquero et al., 2003).

If adolescent and adult sex offending are distinct phenomena, we need to know more about what explain their occurrence at different developmental stages. This research seeks to identify essential differences between the presumably small group of people who continue a criminal career from an early onset sexual offense charge and the much larger group who do *not* continue a criminal career. How do they differ in terms of life circumstances known to influence the chances of criminal behavior over the life-span (Blokland & Nieuwbeerta, 2005)? As noted, clinical research investigating sex crime perpetrators are often based on what Blokland and Nieuwbeerta (2005) categorize as static theories, operating from an assumption of a fixed and more or less stable propensity for offending. In the case of sexual offending, the presence of sexual deviancy constitutes a central element in this propensity. Importantly, the criminal propensity (sexual deviancy) is assumed to influence the development in other life domains (such as education, work, and marriage), while influence the other way around is not expected. Dynamic theories, such as Sampson and Laub’s (1993) theory of informal social control, allow for influence from life circumstances on criminal behavior. Kruttschnitt and colleagues (2000) investigated the role of formal and informal social control in the discontinuity of crime among sex offenders with results indicating that job stability, although not marriage, influences desistance from sex offending. Typology theories represent a third set of life course theories of crime, in which the influence from life circumstances is assumed to be different among different “types” of offenders (most notably adolescent-limited and life course persistent (Moffitt, 1993)). Typology studies based on sex offending samples have provided insight into different aspects of the criminal career. As shown by Cooley’s (2022) qualitative investigation of the desistance process among persons convicted of contact sexual offending, the subsequent trajectories of those who do not reoffend can be further subdivided into a desisting and a non-reoffending pattern. The desisting pattern was characterised by cognitive transformations from an offending to a non-offending identity. The non-reoffending pattern involved behavioral changes, but neither the cognitive nor affective transformations assumed necessary to be considered a desister from sex offending.

A criminal career approach to sexual offending does not mandate a particular theoretical perspective or framework. Rather, studies investigating the progression of sexual offending over time and its antecedents may be informed by different theoretical perspectives, pertaining to the behavioural dimension in focus.

### Age of Onset - When Does Sexual Offending Start?

Crime onset can be operationalized in different ways, with potentially large impact. When based on self-report, age of onset of sexual offending is on average seven years younger than what appears from officially registered onset (criminal justice or police data), a gap which is considerably more pronounced for people who committed sex offenses with child victims than those who offended against adults (Mathesius & Lussier, 2013). Also, when based on official data, age of onset differs between sex offender types; perpetrators with adult victims are generally charged for a first offense in their late twenties/early thirties, while those with child victims are usually in their thirties/forties (Lussier & Cale, 2013). Within the group of perpetrators with child victims, age of onset varies, as crimes with extra-familial victims are initiated earlier than crimes with intra-familial victims (Marshall et al., 1991; Smallbone & Wortley, 2004). Age of the child victim also seems to play a role; those offending against significantly younger victims are often younger at sexual offending onset than those with victims at their own age (Groth, 1977; Hendriks & Bijleveld, 2004). Currently, the literature suggests that there is a continuum of ages of onset of sexual offending which is broader than the 8–16 year age range often observed in the general criminological literature (Farrington, 2003), and which needs to be better identified and understood (Lussier et al., 2021).

### Characteristics of Young Perpetrators of Sexual Offending

According to Seto and Lalumiere's (2010) review and meta-analysis of variables associated with generalist versus specialist theories of sexual offending, adolescent sexual offending is not sufficiently explained as a manifestation of general antisocial tendencies. Overall, general criminal risk factors such as criminal history, antisocial associates, and substance abuse were less often present in adolescent perpetrators of sexual offenses, while atypical sexual interests and childhood abuse experiences were more often present, when compared to adolescent perpetrators of other crimes. Results from adolescents in the Pathways to Desistance study indicated that adolescent perpetrators of sexual offenses were largely similar to other adolescent perpetrators on general criminogenic risk factors (Fanniff et al., 2017). When differentiating between early (12 years of age or less) and adolescent onset of sexual versus non-sexual offending, Rosa and colleagues (2020) concluded that early onset sex offending is characterised by a specific kind of risk, in which sexual abuse plays a central role, along with familial adversity (including parental imprisonment) and neuropsychological adversity (such as ADHD, irritability). In a prospective study looking at childhood risk factors for onset of sex offending, Lussier and coworkers (2015) investigated a set of socieconomic indicators such as source of income, parental education, ethnicity, and neighbourhood characteristics. Their findings indicate that living in a poor neighbourhood, exposure to socioeconomic adversity, behavioural problems, and more extensive juvenile delinquency are among the risk enhancing factors. Accumulation of risk across domains seems to be an important aspect of early onset sex offending.

### Persistence and Diversity – How do Criminal Careers Progress from an Early Onset?

It is a well-supported finding from the general criminal career literature that early-onset offending is associated with a more persistent, frequent and diverse pattern (e.g., Piquero et al., 2003). In the sexual offending field, this observation has transformed into an inaccurate expectation that today’s young (adolescent) perpetrators are tomorrow’s adult perpetrators of sexual violence (Lussier et al., 2021). This has been partly backed by findings from early studies among adult offenders indicating that adolescent-onset of sexual offending was common (e.g., Abel et al., 1987), while later evidence indicate that this is more an exception than a rule (Marshall et al., 1991; Smallbone & Wortley, 2004), although findings are not consistent across studies (Långström, 2002; Miner, 2002). As discussed by Lussier and colleagues (2021), the idea of continuity in sexual offending from adolescence into adulthood is a strongly held assumption which has influenced policies in the sexual offending field from the 1990s up to the present. In a recent meta-analysis of 158 studies of adolescent perpetrators of sexual offenses, Lussier and coworkers (2024) warned against applying adult strategies for prevention among adolescent perpetrators, as recidivism into sexual crimes was unlikely (weighted pooled mean = .08). Further, the study emphasised the more general criminogenic needs present in the group of adolescent perpetrators of sex offenses, evident in their considerably higher rates of recidivism into general crime (weighted pooled mean = .44).

Although several studies have challenged the assumption of high transition rates between adolescent and adult sexual crime (see Nisbet et al., 2004), the idea lingers on. Part of the reason behind this is the observation that adult antisocial behavior is most often preceded by a history of childhood antisocial behavior, and, as sexual offending may be operationalized as a form of antisocial behavior (Seto & Barbaree, 1997), the continuity expectation in sex offending follows from this. However, it has also been observed that most antisocial children do not go on to become antisocial adults, so continuity and discontinuity exists in parallel, a fact which is often referred to as Robins’ continuity paradox (Robins, 1978). Lussier and Blokland (2014) investigated the validity of Robins’ continuity paradox in sex offending using data from the Dutch 1984 birth cohort. Their results did not support the existence of continuity in sexual offending from adolescence through adulthood but rather indicated that juvenile and adult sexual crime perpetration are two distinct phenomena. In their sample, only 4.5% of those with an adult sexual offending record had a sexual crime related history from adolescence, and only 3% of those with one juvenile sex offense went on to adult sex offending. The results of Chouinard-Thivierge and coworkers (2022) further attest to the lack of continuity in adolescent sex offending. Adolescents who had been adjudicated for a sex offense as juveniles (12–17 years of age), were less frequently involved in offending during early adulthood, both when measured by official data and by self-report. Although the idea of persistence in sex offending (coined by the phrase “once a sex offender, always a sex offender”) has been repeatedly empirically refuted (Harris & Hanson, 2004; Jennings et al., 2015; Zimring et al., 2007, 2009) it lingers on in spite of the fact that continuity in sex offending seem to be “(..) unusual, atypical, and rare (..)” (Lussier et al., 2021, p. 96).

### What are the Risk and Protective Factors Influencing Continuity/Discontinuity in Sex Offending?

From studies of general offending trajectories, the role of both formal and informal social control, such as having a job and a non-criminally involved partner emerge as well documented desistance-supporting factors (Humphrey et al., 2024; Zedaker & Bouffard, 2017). In terms of risk enhancing factors, the systematic review and meta-analysis conducted by Yohros (2023) supports the role of adverse childhood experiences on youth (general) reoffending. A similar impact from childhood adversity on continuity in sexual offending has not been observed (Zgoba et al., 2023).

In this study, we focus on the *onset* of sexual offending by investigating the social and criminal trajectories of juveniles indicted of a sexual crime before the age of criminal responsibility (age 15). From that point of departure, we investigate offending patterns according to persistence and diversity, focusing on the dynamic influence of life circumstances on these career parameters. The current paper has three aims. First, we investigate whether juveniles charged with any sex offense in the period 1997–2005 differ from juveniles charged with either violent offenses or other crimes (non-sexual and non-violent combined) on a set of social indicators. Secondly, we track the sample’s criminal records for a period of 22 years, exploring what kinds of offenses are committed by those within the crime type groups defined above who have a subsequent criminal record, and thirdly, whether any of the social background indicators can distinguish between recidivists and non-recidivists.

## Material and Method

The data for this study is derived from Norwegian administrative data, provided for research purposes by Statistics Norway. Everyone with a resident permit in Norway gets a personal identification number which is used for a wide range of purposes, including the population register (births, deaths, marriages, migration etc.), tax authorities, education system, as well as the police. When data is sent to Statistics Norway for statistical purposes, they can link data across registers, and de-identified data can be made available for research purposes. A more thorough explanation of the Norwegian statistical system is provided by Lyngstad and Skardhamar (2011). Norwegian crime statistics cover all crimes investigated from 1992 up to the present, including information on final judicial decision made against the (suspected) perpetrator. Thus, these data are on all solved crimes where there is a charged person. Since a crime might be solved even if the perpetrator cannot be convicted, the data includes persons also under the age of criminal responsibility, which is 15 years in Norway.

### Setting

The age of criminal responsibility varies markedly between countries, as does the range of sanctions available for the youngest perpetrators. In Norway, perpetrators younger than age 15 can be charged, but the case is dropped from further legal prosecution due to lack of criminal responsibility. When closing the case due to lack of criminal responsibility, the police can decide to transfer the case to the child protection services (according to §71b in the Criminal Procedure Act). The child protection services then decide if and what kind of further measures are needed. The criminal charge and the reason for closing the case (perpetrator not criminally responsible) remain in the police registers with details on the charged perpetrator. Information related to if and how other service providers follow-up on the case is not included in the police register.

### Study Sample

The sample used for analyses is established from the population registers and the police registers retrieved from Statistics Norway. The sample used in the current study consists of the total population birth cohorts aged 15 during the period 1997–2005 (*N* = 388,814), of which 19,559 had criminal charge before their 15^th^ birthday. Since relatively many juveniles commit crimes at some point during adolescence, the risk of recidivism should be compared with the risk of offending in this age group, thus we include also those with no offences before age 15 (*n* = 369,255). Those with a recorded crime before age 15, are sorted into the following groups:

(a) Any sex offense covered by regulations in Chapter 26 of the Norwegian penal code. This includes offenses in three broadly defined categories: unwanted sexual behaviours (involving exposure to pornography, indecent exposure), unwanted sexual acts (such as being touched, being forced to masturbate someone) and unwanted penetration/intercourse (including rape).
(b) Any non-sexual violence, including all violence against person (including manslaughter and murder), threats, and violence against public servant.
(c) All other crimes (mainly drug and property crimes, but also economic crimes and damage to property).

In the case of a person having committed multiple types of crimes, these categories are applied hierarchically so that those classified as non-sexual violent offenders have not been charged with any sexual offences, and other offenders have no sexual or violent offences.

### Measures

*Family income (in total)* – defined as household income from work, measured in Norwegian currency at the juvenile’s age 15. Adjusted for consumption units according to the EU scale.^1^

*Family type* – defined as households with children, containing either one or two adults (parents).

*Place of residence* – defined as juvenile living in Oslo (the capital city) at age 15 or not.

*Parents’ educational attainment* – defined as the highest attained level of education achieved by either parent at the juvenile’s age 16.

*Immigrant background* - defined according to the definition used in the population register, as having immigrated themselves or being the descendant of two immigrants (parents born outside of Norway).

*Parental criminal involvement* – defined as any criminal charge raised against the mother/father when the juvenile is 10–15 years old.

*Recidivism* - defined as a new, recorded offence, committed after the initial charge. As we are interested in types of offence committed when recidivating, our outcome measure is re-arrest, as this includes information on all offences, not only the primary/index offence. The police data include dates for both when the crime was solved, reported and committed. In all analyses, we use the date when the crime was committed. Thus, the time to recidivism is less affected by e.g., investigation time, only if the crime is solved within the observation window.

### Analyses

Recidivism analyses often focus on whether an offender re-offends during a given period, but this can be problematic when there is varying observation time such as in our data. Thus, we use survival analysis to take varying exposure time into account. We use Kaplan-Meier plots to show the baseline risk of recidivism as a function of time (months), conditional upon still being at risk. We use proportional hazard models (Cox regression) to take other characteristics into account. In Cox regression the outcome is the risk of an event at time *t*, conditional on being at risk at that time, which is denoted the hazard rate. Thus, the regression coefficients are comparison of hazard rates for groups, and the exponent of the regression coefficients, exp (*β*) are interpretable as hazard ratios (HR).

One disadvantage of survival analysis is that its main purpose is for analysis of time to a single event or competing risk. In our context, the types of recidivism are not mutually exclusive for several reasons. One can commit multiple types of crimes at the same occasion, and one might commit multiple repeated crimes of varying types. For this reason, we estimate separate models with time to selected recidivism types. We first run Cox regression models where the outcome is time to a new crime of any type. We then proceed to repeat the analysis for the outcome of any sexual crime, while ignoring any other types of crimes. Finally, we repeat the analyses with the outcome of any non-sexual violent crime, ignoring any other types of crimes.

For each outcome, we present two Cox regression models. The first only includes the characteristics of main interest: criminal history before age 15. The second model includes several basic control variables: sex, type of family household, parental educational level, immigrant background, resident in major city, and whether at least one parent has a criminal record. These controls capture basic social background characteristics which are correlated with early onset of crime. The estimates for the variables capturing criminal history before age 15 are thus likely reduced when taking these into account.

While it is common to measure recidivism as a single event in this way, criminal career research has for a long time emphasized the need to distinguish between persistent offenders and more time-limited patterns of offending. These sequences of repeatable offending are sometimes summarised using latent class growth curves (Nagin & Land, 1993), which cluster individuals with similar offending patterns into a specified number of groups and estimates their average trajectory as a nonlinear function. One of the disadvantages of that method is that it is primarily suited for studying one kind of outcomes at a time. In our case, it is more relevant to study the sequence of alternating types of crimes, in particular sexual, violent and non-violent crimes. To do so, we use sequence analysis, which has gained popularity in life course research (Liao et al., 2022) and have a similar goal of summarising trajectories into a manageable number of groups using nonparametric methods.

A sequence are events distributed over discrete time intervals, such as years. For one individual, the sequence might consist of sexual crime, violent crime or other types of crimes, with periods of no crime in between. With ten time periods, a sequence for an individual might i.e., look something like {N-N-O-N-V-O-N-N-S-N}, where each position represents either if no crime (N) occurred in that time interval, or if violent crime (V), sexual crime (S), or other crimes (O) occurred. A non-recidivist would accordingly only have N’s at all periods.

The individuals’ sequences are then reduced into clusters of people with similar offending patterns over time. We use the hierarchical agglomerative clustering algorithm with Ward’s aggregation criterion (Müllner, 2013). Each individual is then classified into the cluster he/she resembles the most. Sequences with lack of variation gives little substantive sense and leads to computational problems. Thus, the clustering of sequences is only done on those who have recidivated more than once. The final groups for analysis contain the non-recidivist and single recidivists in addition to the clustered sequences. We then further explore the risk factors of criminal careers using these groups as the outcome variable in multinomial logistic regression models, where the exp (*β*) is interpretable as an odds ratio (OR).

Sequence analysis is a data driven exploratory approach, but there are choices to be made by the researchers that are likely to affect the results, but there are no clear-cut rules for these choices. First, one has to decide on how the individual sequences are constructed. We use yearly periods because finer time intervals of relatively rare events yield sequences which are mainly non-offending and gives little variation to analyse. Since persons might commit multiple crimes in the same year, we construct the outcome similarly as the initial categories of criminal history before age 15: having committed (1) any sexual crime, (2) any non-sexual violent crime, (3) any non-violent crime, or (4) any other crime excluding sexual or violent crimes. Thus, a period with sexual crime does not rule out that other crime types have been committed as well, but any period of “other crimes” do not contain violence or sexual violence. Sequence analysis is mainly meaningful for the part of the sample where there is variation over time. Additional technical details are provided in the Appendix.

Finally, we use the clusters from sequence analysis as outcome variable in a logistic regression analysis, using the same predictors as in the survival analysis.

Data has been provided from Statistics Norway, reference no. 21/3982 in accordance with Norwegian regulations. Data Protection Impact Assessment (DPIA) has been created in agreement with the Data Protection Official at the University of Oslo and filed with Norwegian centre for research data, project no 436491. The authors take responsibility for the integrity of the data, the accuracy of the data analyses, and have made every effort to avoid inflating statistically significant results.

## Results

During the period 1997–2005, the Norwegian population consisted of a total of 388,814 juveniles 15 years of age. Of those, 19,559 juveniles (5%) were charged with a crime. For the majority of those charged (83%), the index crime was non-violent. For the remaining 16.9% (*n* = 3,309), the charge involved non-sexual violence (*n* = 2,991, 15.3% of those charged) or sexual violence (*n* = 318, 1.6% of those charged). In Table 1 the background characteristics of these subgroups are further described.

**Table 1.** Descriptive Statistics by Registered Crimes Before Age 15

Characteristic	Overall, *N* = 388,814	Not offended before age 15, *N* = 369,255	Sexual violence, *N* = 318	Violence, *N* = 2,991	Other, *N* = 16,250
Charged with new offence, %					
No recidivism	85.4	87.2	32.7	30.8	54.2
Sexual violence	0.7	0.5	18.2	3.5	2.2
Violence	2.3	2.1	7.2	7.3	4.8
Other	11.7	10.1	41.8	58.4	38.9
Gender					
Female	48.7	49.6	4.1	19.0	33.3
Male	51.3	50.4	95.9	81.0	66.7
Age at first offence					
Mean (SD)	13.2 (1.6)	-	13.0 (1.7)	13.3 (1.5)	13.2 (1.6)
No of charges before age 15					
Mean (SD)	0.1 (0.7)	-	2.4 (3.4)	3.0 (4.8)	1.6 (1.8)
Parents’ educational attainment, %					
Primary school or less	12.7	12.1	33.6	31.7	22.0
Secondary school	47.8	47.7	44.0	46.0	49.7
University level	39.5	40.2	22.3	22.3	28.4
Family type, %					
Two adult household	98.8	99.0	93.1	94.3	96.5
One adult household	1.2	1.0	6.9	5.7	3.5
Immigrant background, %	6.4	6.1	13.8	21.3	10.4
Family income from work (EU-scale) in 1,000 NKr					
Mean (SD)	232 (177)	235 (178)	163 (142)	163 (142)	190 (158)
Capital city residency, %					
No	91.8	92.0	89.3	80.7	88.4
Yes	8.2	8.0	10.7	19.3	11.6
Parental criminal involvement, %					
No	95.0	95.5	86.5	81.0	87.4
Yes	5.0	4.5	13.5	19.0	12.6

As seen from Table 1, the gender distribution is more skewed for sexual crimes than for other crimes, with boys constituting 96% of those being charged. Boys charged with sexual crimes are generally younger and have more charges against them than the other groups. Both groups charged with violence (sexual and non-sexual) share a more problematic family background: low parental education, lower income, more one-adult households, and more parental involvement in crime. Juveniles with immigrant background are overrepresented in all crime-involved groups, with the highest proportion (21%) among those charged with violent crime.

**Figure 1.**
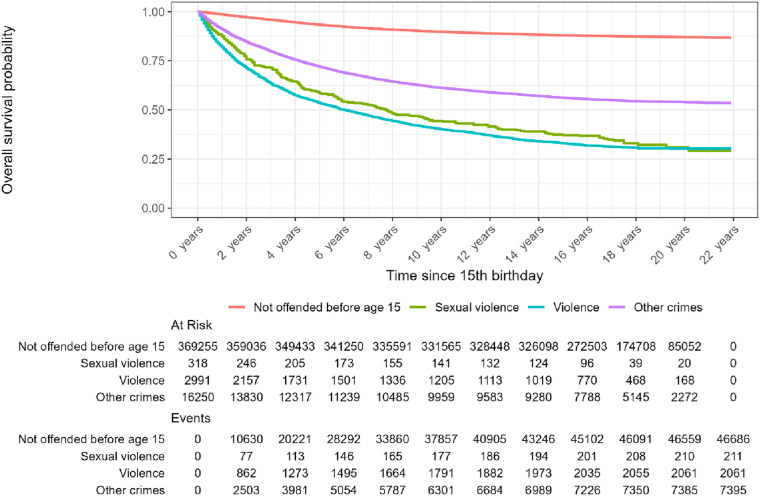
Kaplan-Meier-plot. Time to General Recidivism, by Crimes before Age 15. *Note.* The Table Under the Graph Gives the Population at Risk and the Cumulative Events up to and including the Indicated Time Points on the X-Axis

**Figure 2.**
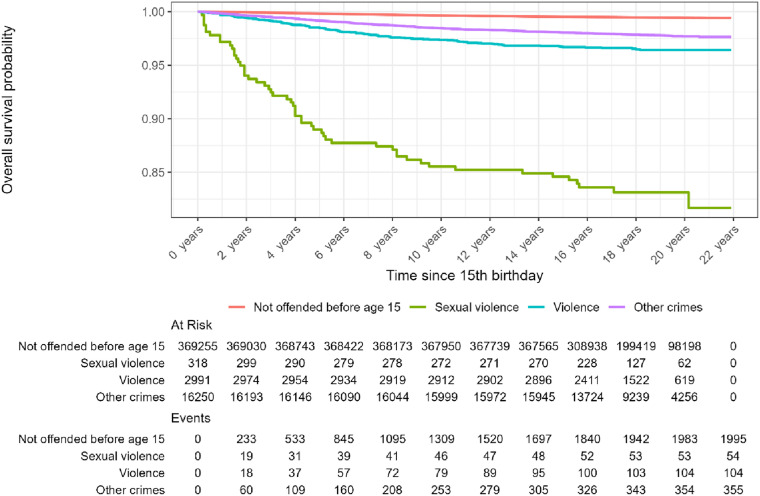
Kaplan-Meier-plot. Time to Recidivism to Sexual Violence, by Crimes before Age 15. *Note.* The Table Under the Graph Gives the Population at Risk and the Cumulative Events up to and including the Indicated Time Points on the X-Axis

**Figure 3.**
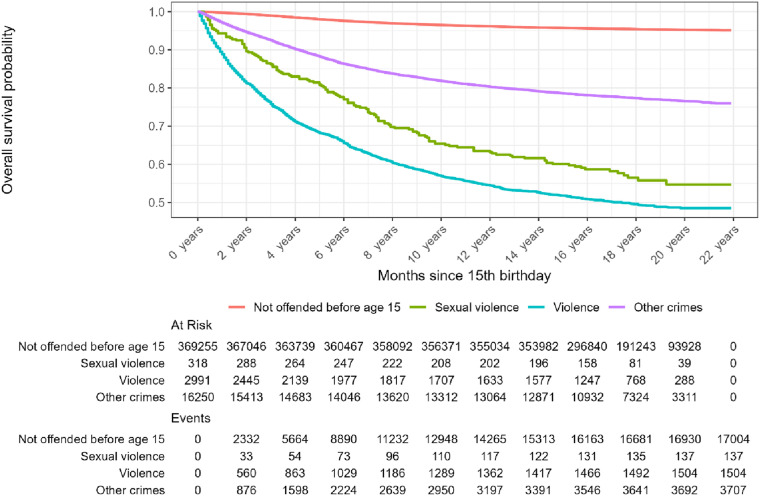
Kaplan-Meier-plot. Time to Recidivism to Violence, by Crimes before Age 15. *Note.* The Table Under the Graph Gives the Population at Risk and the Cumulative Events up to and including the Indicated Time Points on the X-Axis

Our second aim related to persistence of crime among juveniles having received a criminal charge before age 15. This was investigated by tracking the criminal records among the three groups and those without offending before age 15, as illustrated by Kaplan-Meier plots of time to general recidivism (Figure 1), sexual recidivism (Figure 2) and violent recidivism (Figure 3).

Figure 1 shows the time to *first offence* of any kind after age 15, by what kind of offending they had committed before age 15. The time scale is measured as months since 15^th^ birthday. As expected, those without criminal charges before age 15 had consistently lower risk of committing an offence during the follow-up period, as illustrated by the fact that only 13% had received a criminal charge by age 37 (22 years after their 15^th^ birthday). Those with violent or sexual charges prior to age 15 had similar and considerably higher recidivism risk (69% and 66%, respectively) than those with other criminal charges (46%).

In Figure 2, time to first *sexual* offense (ignoring other types of crimes) after age 15 is presented. The scale of the *y*-axis is changed in order to make differences visible. It should be noted that sexual offenses are a relatively rare type of offending, constituting only 4.5% of all registered charges during the follow-up period. Those with previous sexual violence is a very small group (*n* = 318), but their risk of sexual recidivism is high (17%) compared to the other groups. The recidivism occurs mainly the first five years (about age 20) and then the rate levels off. Among the remaining groups, those with early violent charges have a higher risk of sexual recidivism (3.5%) than the other groups. The largest share of sexual recidivism is attributable to persons previously charged with violence.

Figure 3 presents time to first *violent* offense after age 15. The group with previous violence holds the highest risk of violent recidivism (50%), followed by those with previous sexual offenses (43%). Table 2 presents regression coefficients, *β*, with 95% confidence intervals, *CI*, from Cox regression models of time to recidivism.

**Table 2.** Cox Regression, by Type of Recidivism. Regression Estimates With Confidence Intervals

Outcome	Model 1	Model 2	Model 3	Model 4	Model 5	Model 6
	All crimes	All crimes	Sexual violence	Sexual violence	Violence	Violence
Crime before age 15						
Sexual violence	2.08	1.43	3.57	2.64	2.48	1.69
	[1.94, 2.21]	[1.29, 1.56]	[3.30, 3.84]	[2.37, 2.91]	[2.31, 2.65]	[1.52, 1.86]
Violence	2.19	1.62	1.88	1.12	2.76	2.08
	[2.15, 2.24]	[1.58, 1.67]	[1.69, 2.08]	[0.92, 1.33]	[2.71, 2.81]	[2.02, 2.13]
Other crimes	1.52	1.22	1.40	0.93	1.71	1.32
	[1.49, 1.54]	[1.19, 1.24]	[1.29, 1.52]	[0.82, 1.05]	[1.67, 1.75]	[1.28, 1.36]
Male		1.06		3.74		1.53
		[1.04, 1.08]		[3.47, 4.02]		[1.50, 1.57]
Log of family income adjusted (EU-scale)		−0.04		−0.05		−0.04
		[−0.044, −0.038]		[−0.06, −0.03]		[−0.04, −0.03]
One adult household		0.15		0.50		0.25
		[0.10, 0.21]		[0.28, 0.71]		[0.18, 0.33]
Parents: Secondary school		−0.31		−0.33		−0.33
		[−0.33, −0.28]		[−0.44, −0.23]		[−0.36, −0.30]
Parents: University level		−0.73		−0.89		−0.87
		[−0.75, −0.70]		[−1.01, −0.76]		[−0.92, −0.83]
Immigrant background		0.20		0.05		0.36
		[0.17, 0.23]		[−0.10, 0.20]		[0.32, 0.41]
At least one parent with criminal record		0.56		0.27		0.60
		[0.54, 0.59]		[0.14, 0.41]		[0.56, 0.64]
Resident capital city		0.08		−0.42		−0.01
		[0.05, 0.11]		[−0.60, −0.25]		[−0.06, 0.04]
Num.Obs.	388,814	388,814	388,814	388,814	388,814	388,814
AIC	1,424,050	1,402,160	63,415	60,313	561,227	547,048
BIC	1,424,083	1,402,279	63,448	60,432	561,260	547,167

The first two models present risk of *any* crime after age 15, using those without prior charges as the reference group. Those with violent priors have the highest risk of recidivism (*β* = 2.19, *CI* = [2.15, 2.24], HR = 8.9), followed closely by those with sexual offences (*β* = 2.08, *CI* = [1.94, 2.21], HR = 8.0), reflecting the observation from Figure 1, although slightly overlapping confidence intervals. An elevated risk remains after controlling for sociodemographic background in the second model, although the difference between violent and sexual offenders increases after controls (*β* = 1.62 vs 1.43, and HR = 5.1 vs 4.2).

The second set of models estimates the risk of sexual offenses. Those with prior sexual offences have a particularly elevated risk of sexual recidivism (*β* = 3.57, *CI* = [3.3, 3.84], HR = 35.5) compared to the other offense groups ( $\beta_{violence}=1.88$ , CI = [1.69, 2.08], HR_*violence*_ = 6.6 and $\beta_{other\;crimes}=1.4$ , CI = [1.29, 1.52], HR_*other crimes*_ = 4.1). This difference in risk is substantially reduced, however, when controlling for background characteristics (*β* = 2.64 vs 1.12 and 0.93, HR = 14.0 vs 3.1 and 2.53). The third set of models estimates the risk of violent crimes. Those previously charged with violence carry the highest risk for violent recidivism (*β* = 2.76, *CI* = [2.71, 2.81], HR = 15.8). The risk is also considerably elevated among those with prior sexual offenses (*β* = 2.64, *CI* = [2.31, 2.64], HR = 14.0). When controlling for background characteristics, the risk is reduced, while the difference between groups increases. Not surprisingly, male gender is the most consistent contributor to increased risk across crime types, particularly so for sexual recidivism (*β* = 3.74, *CI* = [3.47, 4.02], HR = 42.1). The background characteristics generally contributed in the expected direction, although the effects were not always statistically significant. One adult household elevates the risk of crime generally (*β* = 0.15, *CI* = [0.1, 0.2], HR = 1.2), for sexual violence (*β* = 0.5, *CI* = [0.28, 0.71], HR = 1.65) and violence (*β* = 0.25, *CI* = [0.18, 0.33], HR = 1.3). Having at least one parent with a criminal record increase risk of crime (*β* = 0.56, *CI* = [0.54, 0.59], HR = 1.8), sexual violence (*β* = 0.27, *CI* = [0.14, 0.41], HR = 1.3) and violence (*β* = 0.60, *CI* = [0.56, 0.64], HR = 1.8), while immigrant background is not a significant contributor to sexual recidivism (*β* = 0.05, *CI* = [-0.1, 0.2], HR = 1.1), but increases risk of crime generally (*β* = 0.2, *CI* = [0.17, 0.23], HR = 1.2) and violence (*β* = 0.36, *CI* = [0.32, 0.41], HR = 1.4).

**Figure 4.**
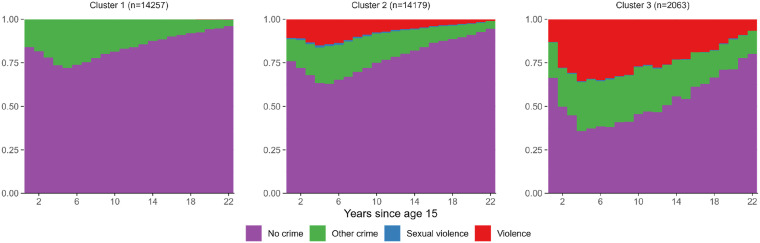
Clusters of Sequences

Time-to-event analysis considers only time to first new offence, while it is equally important to consider the further criminal career of those with criminal charges at a young age. This can be obtained by clustering sequences of events, as presented in Figure 4.

The sequence analysis resulted in four main clusters as displayed in Figure 4. All clusters exhibit mainly non-offending (purple), but different occurrences of violent (red), sexual (blue) and other (green) offending. In cluster 1 the crimes committed are almost exclusively of non-violent kinds. Cluster 2 resembles cluster 1 but has a slightly higher prevalence of crime, and there is more violence. Sexual offenses are relatively rare in all clusters, but is most primarily concentrated in cluster 2. Cluster 3 has a substantial amount of violence along other types of crimes, but less sexual violence than cluster 2. Thus, cluster 1 represent the mainly nonviolent offenders, while clusters 2 and 3 captures violent offenders and separates reasonably well between mainly violent offenders and a group of combined violent and sexual offenders.

Further descriptives of the clusters are presented in Table 3, where also the remaining sample is included.

**Table 3.** Descriptive Characteristics of Crime Clusters

	Overall	Not clustered	Cluster 1	Cluster 2	Cluster 3
N	65,385	34,886	14,257	14,179	2,063
Number of crimes after 15					
Mean	6.49	1.36	7.31	13.08	41.86
Number of sexual crimes after 15					
Mean	0.075	0.035	0.001	0.247	0.097
Total number of crimes	4,919	1.204	11	3,504	200
Number of violent crimes after 15					
Mean	0.97	0.23	0.02	2.49	9.64
Total number of crimes	63,314	7,836	276	35,320	19,882
Number of other crimes after 15					
Mean	5.45	1.10	7.29	10.34	32.12
Males	74.6%	67.2%	77.0%	87.6%	94.5%
Age at first offence, mean (SD)	13.2 (1.6)	13.3 (1.6)	13.3 (1.6)	13.2 (1.6)	13.1 (1.6)
No of charges before age 15, mean (SD)	0.4 (1.5)	0.1 (0.6)	0.4 (1.6)	0.7 (2.2)	1.6 (3.9)
Parents’ educational attainment					
Primary school or less	21.2%	17.4%	22.8%	27.2%	33.1%
Secondary school	50.9%	50.9%	51.0%	51.1%	48.6%
University level	27.9%	31.7%	26.3%	21.8%	18.3%
Family type					
Two adult household	97.0%	98.1%	96.8%	95.3%	92.1%
One adult household	3.0%	1.9%	3.2%	4.7%	7.9%
Immigrant background	9.7%	8.1%	9.1%	13.3%	15.0%
Family income from work (EU-scale) in 1,000 NKr. Mean (SD)	193.60 (147.32)	209.33 (150.97)	185.57 (149.28)	169.94 (133.31)	145.69 (124.81)
Capital city residency	9.5%	9.0%	9.4%	10.6%	11.2%
Parental criminal involvement	10.4%	7.9%	11.7%	14.0%	20.2%

As seen from Table 3, the clusters differ in terms of number of crimes. The largest cluster, Cluster 1, is the low-prevalent group, although the mean number of crimes is perhaps surprisingly high (mean = 7.31). The numbers of sexual and violent crimes are low.

Cluster 2 have a higher number of crimes (mean = 13.1), and also more violent crime (mean = 2.5). As sexual violence is relatively rare, the average number is not high in absolute terms (mean = 0.2), but is far higher than in cluster 1 (mean = 0.001) and cluster 3 (mean = 0.1) Cluster 3 has a higher prevalence of violence (mean = 9.6) in addition to other types of crimes (mean = 32.1). The gender distribution is expectedly skewed, with clusters 2 and 3 containing the largest share of males. Cluster 3 appears to be the most vulnerable of the groups; more charges at a young age, more violence, and coming from a family with lower educational level, lower incomes, more often a one adult household, more often a parent with criminal record, and more often having immigrant background.

In Table 4, results from multinomial regression analyses of likelihood of belonging to each cluster are presented.

**Table 4.** Multinomial Logistic Regression for Cluster Membership From Sequence Analysis. Regression Coefficients and Confidence Intervals

	Model 1 (ref = not clustered)			Model 2 (ref = not clustered)		
	Cluster 1	Cluster 2	Cluster 3	Cluster 1	Cluster 2	Cluster 3
(Intercept)	−1.00	−1.13	−3.32	−0.79	−1.43	−4.09
	[−1.02, −0.98]	[−1.15, −1.11]	[−3.38, −3.26]	[−0.89, −0.69]	[−1.54, −1.33]	[−4.34, −3.84]
Crime before age 15 (ref = none)						
Sexual violence	0.78	2.09	2.29	0.58	1.72	1.77
	[0.34, 1.21]	[1.75, 2.44]	[1.71, 2.86]	[0.14, 1.02]	[1.37, 2.07]	[1.18, 2.35]
Violence	0.93	2.05	3.20	0.81	1.82	2.88
	[0.79, 1.07]	[1.93, 2.17]	[3.05, 3.36]	[0.67, 0.95]	[1.69, 1.94]	[2.72, 3.04]
Other crimes	0.80	1.18	1.75	0.73	1.06	1.57
	[0.74, 0.86]	[1.13, 1.24]	[1.64, 1.86]	[0.67, 0.79]	[1.00, 1.12]	[1.46, 1.69]
Male				0.52	1.27	2.16
				[0.47, 0.56]	[1.21, 1.32]	[1.97, 2.35]
Log of family income from work (EU-scale) in 1,000 NKr				−0.04	−0.04	−0.07
				[−0.04, −0.03]	[−0.05, −0.03]	[−0.08, −0.05]
One adult household				0.02	0.29	0.43
				[−0.12, 0.16]	[0.16, 0.43]	[0.20, 0.66]
Parents: Secondary school				−0.18	−0.25	−0.37
				[−0.24, −0.13]	[−0.31, −0.20]	[−0.48, −0.26]
Parents: University level				−0.34	−0.59	−0.77
				[−0.39, −0.28]	[−0.65, −0.53]	[−0.91, −0.63]
Immigrant background				−0.02	0.31	0.26
				[−0.09, 0.06]	[0.24, 0.38]	[0.11, 0.40]
At least one parent with criminal record				0.32	0.43	0.69
				[0.25, 0.39]	[0.37, 0.50]	[0.57, 0.82]
Resident in the capital city				−0.01	−0.04	−0.13
				[−0.08, 0.07]	[−0.12, 0.03]	[−0.29, 0.02]
Num.Obs.	65,385			65,385		
AIC	140,738.6			136,496.5		
BIC	140,847.6			136,823.7		
RMSE	0.39			0.38		

The first three columns present the results from model 1, showing that prior offences increase the log odds ratio of belonging to each of the clusters 1 through 3, compared to those without prior offences. Those with prior sexual offenses have the highest risk of belonging to cluster 2 (*β* = 2.29, *CI* = [1.75, 2.44], OR = 9.9), and similar risk of belonging to cluster 3 (*β* = 2.29, *CI* = [1.71, 2.86], OR = 9.9). Those with prior violent offenses have similar risk of belonging to cluster 2 (*β* = 2.05, *CI* = [1.93, 2.17], HR = 7.8). Thus, it seems violent and sexual offending before age 15 give similar risk of belonging to these clusters of criminal careers. It is notable that controlling for background characteristics (model (2) does not substantially change the parameter estimates despite some minor adjustments to the parameters.

## Discussion

Based on unselected population registry data, the present study has explored the subsequent criminal careers among persons charged with sexual offending during childhood, prior to the age of criminal responsibility. This small group of predominantly young boys was characterized by social adversity along several dimensions. Children with sexual offense charges emerged as more similar to than different from children charged with other forms of violence, in terms of a vulnerable socioeconomic background: low parental education, lower family income, immigrant background, more one-adult households, and more parental involvement in crime. Taken together, these characteristics reflect an accumulation of risk related to unpriviledged socioeconomic positions. Those charged with sexual offenses were generally younger and had more charges against them than children charged with any other crimes. Early onset of offending turned out to be a notable indication of future offending. Children initiating sexual or violent crime at an early age had substantially elevated risk of general recidivism as well as violent recidivism and sexual violent recidivism compared to the other groups. In particular, what characterises those with early sexual offense onset is their elevated risk of progressing into a serious criminal career later on, with high prevalence of violent crimes.

According to Lussier and Blokland (2014), juvenile and adult sex offending are two distinct phenomena, with little continuity. Our findings support this, as the majority of adult sexual criminal charges are raised against persons without a childhood criminal record (see Figure 2). However, Lussier and colleagues (2014) do note that this overall pattern of discontinuity hides the fact that there is also a very small subgroup of young sexual offense perpetrators who are at high risk of continuity into adult crime. Our findings also demonstrate this: among the 318 individuals charged with a sexual offense before the age of criminal responsibility, 67% have a subsequent criminal record, which is dominated by (non-sexual) violence. It should be emphasized that intervening to abort the transition from childhood criminal involvement to adult crime in this vulnerable subgroup of children is important in its own right. However, framing interventions targeting this small group as a way to prevent adult sexual offending generally, is not empirically substantiated. Our results indicated that eight out of ten sexual offenses committed after age 15, had perpetrators not previously registered with any criminal charges, thus being unknown to the criminal justice system. This underscores the argument made by those arguing for a shift in attention when it comes to preventing sexual harm, from tertiary initiatives to a public health approach (see McCartan et al., 2018).

The prediction models indicated that socioeconomic background variables all contributed to risk of recidivism, thus constituting relevant targets for crime preventive interventions. The importance of family background variables as childhood risk factors for life-course persistent crime is well known (Farrington, 2020). Childhood onset of sexual offending seems to have similar background risk factors as early onset offending generally, as also noted by Mathesius and Lussier (2013). What seems to emerge most consistently is the importance of accumulated risk across multiple domains, as also pointed out by Lussier and colleagues (2015). In the current study, immigrant background serves as an illustration of this point: immigrant background did not remain a significant contributor to sexual recidivism in multivariate models when other indicators of marginalization were included.

## Limitations

The present study presents results from a context outside North America, as called for by Lussier and colleagues (2024). Important as between-countries’ comparisons are, they also come with some limitations. First of all, legal regulations regarding age of criminal responsibility vary considerably between countries, as do the sanctioning system available for those below the age of criminal responsibility. Our Norwegian sample consists of children who have not been subject to any form for *punishment* for their criminal actions (due to lack of criminal responsibility), while in the Netherlands, for instance, children as young as 12 are penalised under juvenile criminal law, as the age of criminal responsibility is 12. The potential consequences of this on risk for recidivism must be taken into consideration when comparing registry data covering the same age groups from different countries. Children included in the studies of Lussier and Blokland (2014) and Lussier and coworkers (2015) are actually criminally sanctioned, and hence subject to a potentially effective crime-preventive intervention. This must be considered when comparing the trajectories of boys aged 13 at first criminal involvement (charge) between these two countries. Generally, it should be noted as a limitation to all studies relying on officially registered crime that a substantial amount of crime is never reported or otherwise officially detected, and this seems to be especially true for sexual offenses. Thus, actual and observed recidivism may be quite different quantities and should not be conflated, as discussed by Scurich and John (2019). Notwithstanding this overall problem with registry-based crime data, the current study has some important strengths. First among these are the high-quality administrative population registers from which the sample is drawn. Every citizen holding a valid personal identification code is included in these registers, and due to the unique personal codes, individuals can be tracked across different registers covering various life domains, and be linked across generations, all of which provides unique opportunities for criminological research (Lyngstad & Skardhamar, 2011).

## Conclusion

Ten years ago, a paradigm shift was called for, to align societal responses to youth sexual offending with sound empirical evidence (Lussier, 2017). American policies and practices, in particular, were critized for being based on myths and unsubstantiated claims about youth sexual offending (Chaffin, 2008; Letourneau & Miner, 2005). A common theme among the critics was the assumption of high risk of recidivism if left untreated, and continuity in sex offending from adolescence into adulthood. The current study adds some nuances to this discussion, based on findings from a different legal and societal context. Although the big picture is one of discontinuity, continuity also exists, albeit for a small and vulnerable subgroup. Evidence-based interventions need to balance these two facts in order to be optimally crime preventing.

## Note

1. See definition at Statistics Norway: https://www.ssb.no/en/inntekt-og-forbruk/inntekt-og-formue/statistikk/inntekts-og-formuesstatistikk-for-husholdninger.

## Appendix

### Details on Sequence Analysis

The sequence analysis in this paper is employed as an exploratory approach to cluster individuals based on the similarity of their offending behavior. This method summarizes trajectories in a way that highlights the patterns and differences among these behaviors.

The basic “distance” between two sequences is based on the minimum number of substitutions in the sequence to make them equal. For example, for two persons with identical sequences except from in one single time point, it would take one substitution to make these sequences equal, thus the distance is 1. However, not all substitutions are necessarily equal, and if so, the distance depends on the type of substitution. This is referred to as substitution costs, and can be assigned in several ways. These substitution costs represent the relative weight of changing one type of offending behavior to another, reflecting the varying degrees of similarity between offenses. We constructed a substitution cost matrix manually as follows:

$$substitution\;cost=\left[\begin{array}{llll} 0 & 1 & 3 & 3\\ 1 & 0 & 3 & 3\\ 3 & 3 & 0 & 2\\ 3 & 3 & 2 & 0 \end{array}\right]$$

where the first row/column indicates substation costs for: no crime, other crimes, sexual violence and other violence. The intention is to separate violence and sexual violence from other crimes.

In addition, the distance metrics can emphasize other characteristics of the sequence such as durations, timing and gaps. Since crime are events rather than states with durations, it is more reasonable to calculate distance metrics in a way that put emphasis on both *timing* and time *gaps* between states, thus we use the Time-Weighted Edit Distance (TWED), (Studer & Ritschard, 2016). We thus include small penalties to the transition costs for timing, ν=0.01 , and time gaps, λ=0.05 .

In our case, there are also unequal follow-up times, thus unequal sequence lengths. This is accounted for by specifying missing values and the distance metrics are normalized to sequence length.

The distance metrics are used for cluster analysis to create clusters of similar trajectories. All steps of the analysis were done using *R* (R Core Team, 2024) and the packages *TraMineR* (Gabadinho et al., 2011). We use the hierarchical agglomerative clustering algorithm with Ward’s aggregation criterion as implemented in the *fastcluster* package (Müllner, 2013).

The results are sensitive to the specification of the model parameters indicated above. Since there is a very large number of potential specifications, this is an exploratory approach and must be interpreted as such. With the aim of finding as clean-cut clusters as possible, we fitted many models while modifying the cost matrix, and the values for ν and λ , but we have not conducted a systematic grid-search for best-fitting model based on statistical criteria. Other types of distance metrics (e.g., optimal matching and principal components coordinates) were attempted, but the model search was restricted to TWED as we considered it the most appropriate for these data. Thus, the final model is based on a combination of statistical criteria and a theoretically informed consideration of meaningfully distinct cluster differences.
